# Perceived Stress and Stressors among Medical and Dental Students of Bhairhawa, Nepal: A Descriptive Cross-sectional Study

**DOI:** 10.31729/jnma.4911

**Published:** 2020-06

**Authors:** Harleen Bali, Vaibhav Rai, Nitin Khanduri, Rupam Tripathi, Khushbu Adhikari, Binam Sapkota

**Affiliations:** 1 Department of Oral Medicine and Radiology, Kathmandu University School of Medical Sciences, Dhulikhel, Kathmandu, Nepal; 2 Department of Oral Medicine and Radiology, Universal College of Medical Sciences-College of Dental Surgery, Bhairhawa, Nepal; 3 Department of Pediatric and Preventive Dentistry, Seema Dental College and Hospital, Rishikesh, Uttrakhand, India; 4 Department of Conservative Dentistry and Endodontics, Universal College of Medical Sciences-College of Dental Surgery, Bhairhawa, Nepal; 5 Department of Periodontology and Oral Implantology, B.P. Koirala Institute of Health Sciences, Dharan, Nepal; 6 Department of Prosthodontics, Kathmandu University School of Medical Sciences, Dhulikhel, Kathmandu, Nepal

**Keywords:** *academic performance*, *medical school*, *mental health*, *stress*

## Abstract

**Introduction:**

Medical school is recognized as a stressful environment that may have a negative effect on a student’s academic performance, health, and psychosocial well-being. This could further impact future health professionals' attitudes and compromise patients' care. This study aims to find out various sources of stress for medical and dental students to help prevent many future health problems in a student’s life.

**Methods:**

It was a cross-sectional study done in Universal College of Medical Sciences, Bhairhawa, Nepal, among undergraduate final year bachelor of medicine and bachelor of surgery and third and fourth year (phase I and phase II) dental students, using a questionnaire with Likert’s scale. Data obtained was tabulated and analyzed using analysis of variance.

**Results:**

Results showed that stress during exam 210 (92.9%) and preparation phase 200 (88.5%) stood out as the maximum stressors for our study group. The least stress-causing element was recorded as terms with seniors 45 (19.9%), adjustment with roommates 52 (23.01%), and competing with peers 69 (30.53%). Length of course 187 (82.74%), understanding the course 173 (76.55%), reading several textbooks 171 (75.66%), and work overload 165 (73.01%) amounted to significant stressors.

**Conclusions:**

Stress has a detrimental effect both on health as well as academic performance. The stressors at the campus should be identified and proper coping assistance should be provided to individual students. Systemic efforts are needed to address their concerns and make mental health care easily accessible to them. Counseling and awareness are recommended.

## INTRODUCTION

Medical students world over have been found at risk of psychological stress, mental disorders, and decreased life satisfaction. It is reported that 27% of health professional students develop psychological morbidities during training.^1^ Various stress-related illnesses, including anxiety, depression, suicidal ideations,^2^ somatoform disorders^3^ have been documented among health professional students. It leads to impaired judgments and addiction to substances such as khat chewing, cigarette smoking, and alcohol drinking.^4^

Although this can be traced from various sources, stress plays a precipitating role. Despite the problem of stress widely being acknowledged in health professional training, little attention has been given to it especially by training institutions in developing countries. Intending to groom future health professionals that are resilient, stress during training has to be identified and addressed, we designed a study with a focus on documenting the prevalence of stress and its sources among health professional students.

## METHODS

This descriptive cross-sectional study was conducted among college students studying at Universal College of Medical Sciences, Bhairhawa, Nepal from July to September 2018. The study population consisted of undergraduate medical students. Convenience sampling was used for the selection of the participants. The study population included all the dental students with clinical subjects and final year medical students. Ethical clearance was obtained from the institutional ethical committee. A questionnaire was distributed manually and displayed in the classroom among students of bachelor of medicine and bachelor of surgery (MBBS) (final year) and a bachelor of dental surgery BDS (3rd and 4th-year phases I and II). The students of M.B.B.S (final year) and B.D.S (3rd and 4th-year phases I and II) in medical college who gave consent were included in the study. The students who were absent on the day of data collection were excluded. The sample size was calculated as follows,

n = Z^2^ × (p × q) / e^2^

    = 1.96^2^ × 0.3 × 0.7 / 0.06^2^

    = 0.81 / 0.0036

    = 225

Where,

n = required sample size
p = prevalence, 30%
q = 1-p
e = margin of error, 6%
Z = 1.96 at 95% Confidence Interval

In the present study, the following information was collected from the participants:

I. Personal data: it included general information regarding age, gender, residential area, course, batch, etc.

II. Stress inducing factors:^5^ They were divided into three categories of potential sources of stress.

a. Academic: academic achievements, examinations, and course material, competition among students (Question 2 to 5).

b. Office relationships:^6^ get certificates, scholarships, etc. from the office (Question 6 to 12). c. Social factors:^6^ social aspects in college, relationships in classrooms, parental influences, seniors’ supports, etc. (Question 13 to 15). Each item was scaled (Likert’s scale)^7^ as:

0: no stress,
1: mild stress (sometimes stressful),
2: moderate stress (often stressful),
3: considerable stress (always stressful),
4: extreme stress.

A total score was obtained from summing up the score of each subgroup. Average subgroups of individuals were compared. Data was entered in Microsoft excel sheet and analyzed using statistical package for social sciences (SPSS) software version 20.

## RESULTS

This study included a total of 226 medical students; all with clinical exposure in the field of medicine. Among them, 122 (53.98%) final year MBBS students and 104 (46.02%) BDS students. BDS included 3rd year were 22 (9.73%), 4th year phase I were 53 (23.45%), 4th-year phase II were 29 (12.84%). The total sample included 107 (47.35%) males, and 119 (52.65%) females (Table 1).

**Table 1:** Variables used in the study.

Variables	n (%)
**Sex**	
Male	107 (47.35)
Female	119 (52.65)
**Programme**	
MBBS	122 (53.98)
BDS	104 (46.02)
**Year of study**	
MBBS Final year	122 (53.98)
BDS 3rd year	22 (9.73)
BDS 4th year Phase I	53 (23.45)
BDS 4th year Phase II	29 (12.84)

The score of individual stressors was totaled; and any score 2 i.e. moderate stress (often stressful) and above were considered, the result showed that stress during exam 210 (92.92%) and preparation phase 200 (88.5%) stood out as the maximum stressors for our study group. The least stress-causing element was recorded as terms with seniors 45 (19.91%), adjustment with roommates 52 (23.01%), and competing with peers 69 (30.53%). Though the length of course 187 (82.74%), understanding the course 173 (76.55%), reading several textbooks 171 (75.66%) and work overload 165 (73.01%) amounted to significant stressors, as noted by the majority of participants, none surpassed the stress perceived during exam time (Table 2).

**Table 2:** Stress response to individual stressors.

Stressors	n (%) (score >2)
Length of the course	187 (82.74)
Understanding the course	173 (76.55)
Reading number of textbooks	171 (75.66)
Work overload	165 (73.01)
Keeping in your teachers good books	140 (61.95)
Maintaining good terms with all your batch mates	79 (34.96)
Stress during exams	210 (92.92)
Preparation phase	200 (88.5)
Parental pressure to excel	81 (35.84)
Competing with peers	69 (30.53)
Score well for certificates/ scholarships	146 (64.6)
Being away from home	138 (61.06)
Adjustment with roommates	52 (23.01)
Terms with seniors	45 (19.91)

Among the male and female participants, 118 (99.2%) out of 119 (female participants) reported stress during exam time as compared to 92 (85.04%) out of 107 male participants. 109 (91.6%) females find preparatory phase and length of course as stress giving as compared to 91 (85.9%) and 74 (69.15%) males respectively. Least stress-causing for the female group was having terms with seniors 16 (13.4%) and adjustment with roommates 21 (17.6%) as was for males too, 29 (27.1%), and 31 (28.97%) respectively (Table 3).

**Table 3:** Stress response to individual stressors (Gender wise).

Stressor	Male (score >2) n (%)	Female (score >2) n (%)
Length of the course	74 (69.15)	109 (91.6)
Understanding the course	70 (65.42)	100 (84.03)
Reading number of textbooks	74 (69.15)	98 (82.35)
Work overload	69 (64.48)	96 (80.67)
Keeping in your teachers good books	63 (58.88)	77 (64.7)
Maintaining good terms with all your batch mates	48 (44.85)	31 (26.05)
Stress during exams	92 (85.9)	118 (99.2)
Preparation phase	91(85.04)	109 (91.6)
Parental pressure to excel	46 (42.9)	35 (29.4)
Competing with peers	34 (31.77)	35 (29.4)
Score well for certificates/scholarships	63 (58.88)	83 (69.74)
Being away from home	49 (45.79)	89 (74.78)
Adjustment with roommates	31 (28.97)	21 (17.6)
Terms with seniors	29 (27.1)	16 (13.4)

When comparing subject wise, stress during exam was again the most noted stressor among both MBBS 115 (94.26%) and BDS 95 (91.35%) students; wherein among BDS 3rd year noted 22 (100%), 4th-year phase I and phase II noted 47 (88.68%) and 26 (89.65%) respectively, which was highest among these subgroups too. MBBS students gave second place to preparatory phase 113 (92.62%), while the second high stress-causing factor for BDS students was the length of the course, work overload each scoring 91 (87.5%) where n was equal to or more than 2. Least stress-causing among MBBS were terms with seniors 27 (22.13%) and competing with peers 34 (27.87%). For BDS students low stress-causing factors were tearms with senior 18 (17.31%) with seniors and adjustment with roommates 22 (21.15%) respectively (Table 4).

**Table 4:** Comparison of stress response to individual stressor among MBBS, BDS subject clinical subject-wise and MBBS final year, BDS 3rd year, BDS 4th year phase I and phase II as groups.

Stressors	MBBS Final year n (%)	BDS 3rd year n (%)	BDS 4th year 1st phase n (%)	BDS 4th year 2nd phase n (%)	BDS n (%)
Length of the course	96 (78.68)	20 (90.91)	43 (81.13)	28 (96.55)	91 (87.5)
Understanding the course	92 (75.41)	19 (86.34)	37 (69.81)	25 (86.21)	81 (77.88)
Reading number of textbooks	87 (71.31)	20 (90.91)	38 (71.7)	26 (89.65)	84 (80.77)
Work overload	74 (60.65)	18 (81.82)	47 (88.68)	26 (89.65)	91 (87.5)
Keeping in your teachers good books	67 (54.92)	16 (72.73)	35 (66.04)	22 (75.86)	73 (70.2)
Maintaining good terms with all your batch mates	52 (42.62)	8 (36.37)	15 (28.30)	4 (13.8)	27 (25.97)
Stress during exams	115 (94.26)	22 (100)	47 (88.68)	26 (89.65)	95 (91.35)
Preparation phase	113 (92.62)	19 (86.34)	40 (75.47)	28 (96.55)	87 (83.65)
Parental pressure to excel	53 (43.44)	9 (40.91)	17 (32.07)	2 (6.9)	28 (26.92)
Competing with peers	34 (27.87)	7 (31.82)	20 (37.73)	8 (27.59)	35 (33.65)
Score well for certificates/scholarships	73 (59.84)	15 (68.18)	28 (52.83)	23 (79.31)	66 (63.46)
Being away from home	68 (55.74)	11 (50)	35 (66.04)	24 (82.76)	70 (67.31)
Adjustment with roommates	30 (24.59)	5 (22.73)	12 (22.64)	5 (17.24)	22 (21.15)
Terms with seniors	27 (22.13)	7 (31.82)	6 (11.32)	5 (17.24)	18 (17.31)

One way any of variance (ANOVA) was used to compare means of different stressors in various batches (Table 5).

**Table 5:** Comparison of mean score for Stress response to individual stressor among MBBS students final year, BDS 3^rd^ year, BDS 4^th^ year phase I and phase II.

Stressors	MBBS Final year	BDS 3rd year	BDS 4th year 1st phase	BDS 4th year 2nd phase	F-value
Length of the course	2.4±1.2	3.05±1.2	2.5±1.01	2.72±0.84	2.325
Understanding the course	2.1±1.01	2.3±0.8	2.1±1.02	2.3±0.8	0.732
Reading number of textbooks	2.3±1.3	2.6±0.91	2.3±1.21	2.5±0.9	0.695
Work overload	2.03±1.3	2.5±1.1	3.1±1.2	2.9±1.03	11
Keeping in your teachers good books	1.8± 1.3	1.8±1.1	1.96±1.2	2.34±1.3	1.35
Maintaining good terms with all your batch mates	1.5± 1.5	1.4±1.1	0.98±1.1	0.75±0.91	3.25
Stress during exams	3.3± 0.9	3.6±0.66	3.2±1.3	3.41±0.98	0.907
Preparation phase	3.1±1.01	2.91±1.02	2.36±1.2	3±0.9	5.78
Parental pressure to excel	1.6±1.5	1.1±0.9	1.1±1.1	0.5±0.6	6.5
Competing with peers	1.02±1.3	1.3±0.93	1.4±1.2	1.1±1.1	1.03
Score well for certificates/scholarships	1.9±1.4	2.13±1.3	2.03±1.3	2.3±1.04	0.75
Being away from home	1.8±1.5	1.9±1.6	2.3±1.5	2.6±1.4	3.52
Adjustment with roommates	0.9±1.4	0.7±0.8	1.02±1.3	0.9±1.1	0.367
Terms with seniors	0.9±1.1	1.1±0.8	0.4±0.7	0.6±0.7	3.98

## DISCUSSION

Stress among students is a global phenomenon and studies have revealed that medical students have severe stress levels.^5^ Chronic stress may also lead to deterioration of academic performance, loss of memory, poor relationships with peers and family members, and overall dissatisfaction with life. This chronic exposure can also lead to serious health problems like hypertension, heart attack and stroke, diabetes mellitus, and obesity, accelerated aging, impaired the immune system, suppressed fertility, digestive problems and loss of appetite, and an increases anxiety and depression that finally lead to suicide.^8^ Stress has also been found to be associated with sleep problems and lower academic performances.^9^ Furthermore, it has also been linked to substances use and drug addiction.^10^ Various studies revealed that the stressors affecting medical students’ well-being seems to be related to the medical training especially related to academic matters.^9,11,12^ They found that the top four stressors were tests and examinations, time pressure, too many content to be studied, and getting behind in work. Another three common stressors were conflicting demands, not getting work done within the time planned and heavy work load.^13^

In our study majority of the students experienced stress during exam and preparatory phase, closely followed by academic stressors (length, of course, understanding the course, reading a number of textbooks and work overload). Similar results were reported by Bhavani Nivetha M, et al. in 2018^14^ in their study wherein 40.9% of study participants stated academic-related stressors to be the source of high stress followed by inter and intra-personal related stressors 33.3%. A study done by Gupta, et al. in Kolkatta^15^ showed 94% had academic-related stressors and 78% had Interpersonal stressors. The other studies done by Chowdhury, et al.^16^ Panchu, et al.^17^ Melaku, et al.^18^ all showed a similar result with academic-related stressor being the major contributor of stress.

The vastness of the medical syllabus and lack of proper time management lessons for the students are the main reason for examination time, preparation for it and academic causes being the major stressor.^14^ Yusoff and Esa noted that curriculum differences in medical schools may not necessarily cause differences in the overall pattern of stressors (i.e. most of the top stressors are related to academic matters), although frequency (rank) of some stressors may be significantly different.^10,11,13^

The genesis of emotional and mental stress among medical students may be multifactorial. A medical student has to cope with high expectations of the parents, teachers, and patients; time constraints for pursuing their alternate interests along with trying to keep up with the vast curriculum and exam preparation.^19^ In our study, comparison of means of different stressors in various batches i.e. MBBS final year, BDS 3rd year, 4th-year phase I and phase II showed that work overload, parental pressure to excel, the preparation phase, terms with seniors and being away from home were found to be highly significant. High expectations from self and family members, coupled with the training for assuming responsibility for the well-being of the patient, make a medical student prone to experience stress which may become excessive.^20^

Efforts are required to cater to medical students who are distressed, in a nonintrusive manner. Awareness about manifestations of distress among medical students needs to be increased among not only students themselves, but also other stakeholders such as medical educationists and parents.

It is suggested that each institute should have its orientation program and counseling services. The work should begin from entry to a medical college. A psychiatrist or psychologist present in the interview board at the time of admission, to screen the entrants, will be beneficial.^21^ Survey of US and Canadian medical schools showed 78% of schools had a member of psychiatry faculty in the admission committee. Legislation may vary in different parts of the world, but perhaps those who require assistance and counseling can be identified at the earliest.^22^

Many institutions have employed various techniques for stress management. These include primary preventive measures such as psycho-educational lectures, seminars on stress management, and therapeutic practice like crisis intervention and counseling.^23^ Since resurgence from ‘burnout’ is related with a decline in suicidal tendency, signs and symptoms of this ‘burnout’ should be identified with potential factors which decrease and finally treat it.^2^ Wellness and mental health programs are also needed to help students make a smooth transition between different learning environments with changing learning demands and a growing burden on their mental and physical capacity.^24^ Medical schools in the United States and Canada have initiated health-promotion programs and have reported positive results in reducing the negative effects of stress upon the health and academic performance of medical students.^25^ Medical educators need to know the prevalence, causes, and levels of stress among students which not only affects their health but academic achievements and the well-being of the patient in their care also. With early identification and effective psychological services, possible future illnesses may be prevented. On the other hand, a minimal amount of stress is necessary to add spice to one’s life and to achieve optimal performance at examinations. An element of stress is involved with growth and is essential for sound personal functioning.^24^

This cross-sectional study was based on self-reported information provided by students. Therefore, there is some potential for reporting bias which may have occurred because of the respondents’ interpretation of the questions or desire to report their emotions in a certain way or simply because of inaccuracies of responses. Further long-term studies need to be carried out to investigate the levels of stress among students in all the five years of undergraduate medical years and the associated factors; its relevance to their physical, emotional, and mental wellbeing. Studies could be done to evaluate coping strategies employed by students to overcome stress. Studies focusing on the immediate and long-term impact of stress during medical training on patient care also need to be carried out.

## CONCLUSIONS

It is clear from the results of this study that the student surveyed, were exposed to a variety of environmental and interpersonal stressors. Given the detrimental effects of stress on health and academic performance, there is a need to incorporate stress management training into orientation activities. At a minimum, the most commonly identified sources of stress should be discussed with students. Furthermore, students should be informed of the campus resources available to help them address these stresses. One approach may be the use of stress management workshops. Counseling programs for students to provide effective coping strategies by educationists and support to at-risk students will go a long way during their studies. The presence of a counseling team among the faculty is necessary.

## Conflict of Interest:

**None.**
